# The *Drosophila melanogaster* Y-linked gene, *WDY*, is required for sperm to swim in the female reproductive tract

**DOI:** 10.1101/2023.02.02.526876

**Published:** 2023-02-23

**Authors:** Yassi Hafezi, Arsen Omurzakov, Jolie A. Carlisle, Ian V. Caldas, Mariana F. Wolfner, Andrew G. Clark

## Abstract

Unique patterns of inheritance and selection on Y chromosomes lead to the evolution of specialized gene functions. Yet characterizing the function of genes on Y chromosomes is notoriously difficult. We report CRISPR mutants in Drosophila of the Y-linked gene, *WDY*, which is required for male fertility. *WDY* mutants produce mature sperm with beating tails that can be transferred to females but fail to enter the female sperm storage organs. We demonstrate that the sperm tails of *WDY* mutants beat approximately half as fast as wild-type sperm’s and that the mutant sperm do not propel themselves within the male ejaculatory duct or female reproductive tract (RT). These specific motility defects likely cause the sperm storage defect and sterility of the mutants. Regional and genotype-dependent differences in sperm motility suggest that sperm tail beating and propulsion do not always correlate. Furthermore, we find significant differences in the hydrophobicity of key residues of a putative calcium-binding domain between orthologs of *WDY* that are Y-linked and those that are autosomal. Given that *WDY* appears to be evolving under positive selection, our results suggest that *WDY*’s functional evolution coincides with its transition from autosomal to Y-linked in *Drosophila melanogaster* and its most closely related species. Finally, we show that mutants for another Y-linked gene, *PRY*, also show a sperm storage defect that may explain their subfertility. In contrast to *WDY*, *PRY* mutants do swim in the female RT, suggesting they are defective in yet another mode of motility, navigation, or a necessary interaction with the female RT. Overall, we provide direct evidence for the long-held presumption that protein-coding genes on the Drosophila Y regulate sperm motility.

## INTRODUCTION

Y chromosomes are unique in the genome of many organisms, including mammals and Drosophila, in being haploid, male-limited, repeat-rich, highly heterochromatic, and, in particular, having reduced or no recombination ^1^. The resulting selective pressures on Y chromosomes cause rapid degeneration of most protein-coding genes, yet a few genes are maintained on Y chromosomes with remarkable evolutionary endurance. Such genes are maintained for extended periods under strong purifying or sometimes positive selection, repeatedly and independently acquired in different lineages, or undergo massive copy-number amplification on the Y chromosome ^2–5^. These patterns of variation indicate that selection favors placing such genes into this seemingly inhospitable genomic environment. In support of this concept, there is striking similarity in both the expression patterns and functions of many Y-linked genes ^6–8^.

The 40 MB *Drosophila melanogaster* Y chromosome contains only 14 known protein-coding genes ^9–11^. X0 flies are male yet sterile – therefore, the Y chromosome is required for male fertility but not sex determination or viability ^12^. Six genetic loci on the Y, known as the “fertility factors”, contribute to this fertility function. The fertility factors were defined by a series of X-ray induced X-Y translocations ^13,14^ and, remarkably, half of them were discovered to be axonemal dyneins ^15,16^, suggesting that the *Drosophila melanogaster* Y chromosome plays a pivotal role in sperm motility. *kl-1* males, in contrast to all other fertility factor mutations, produced mature and motile sperm despite being completely sterile^17^. *kl-1* sperm were transferred to the female reproductive tract (RT) following mating but could not be recovered from the female sperm storage organs. The specific defect that prevents *kl-1* mutant sperm from entering storage or fertilizing eggs is unknown. The molecular identity of *kl-1* remained unknown until, recently, the gene, *WDY*, was found to be contained within the *kl-1* region ^18^ and required for male fertility based on RNA interference (RNAi) ^19^. Other protein-coding genes or functional repetitive elements may still reside in the *kl-1* genetic region, which was estimated cytologically to span ~3% of the length of the Y chromosome ^20^, and it is unclear whether *WDY* mutants produce mature sperm or show a sperm storage defect. More generally, the importance of motility for sperm storage and the mechanisms that regulate sperm motility remain poorly understood in Drosophila.

Here we generated CRISPR mutants to investigate the function of *WDY*. We demonstrate that *WDY* mutant sperm display the storage defect suggested for *kl-1.* Furthermore, mutant sperm have reduced beat frequency and are unable to swim beyond the seminal vesicle. We show that mutants in another Y-linked gene, *PRY*, also have impaired sperm-storage. *WDY* and *PRY* are both evolving under positive selection^2^ and Y-linked orthologs of *WDY* show significant changes in key amino acid residues in a conserved calcium-binding domain, suggesting directed functional evolution of this gene.

A high incidence of genes with predicted sperm motility functions are seen on Y chromosomes across many species from Drosophila to great apes ^7,21^. Carvalho et al (2000) hypothesized that, in species where there is a high level of sperm competition (such as *Drosophila melanogaster*), motor proteins are specifically recruited to the Y chromosome where they can evolve without constraint from male-female antagonistic selective forces. Our study provides an in-road to studying the evolutionary logic of this association.

## RESULTS AND DISCUSSION

### *WDY* mutants are sterile but produce mature, motile sperm

We used CRISPR to precisely target *WDY*. One of the major challenges of studying the Y chromosome is in propagating sterile mutations on a haploid chromosome. We used a crossing strategy involving compound sex chromosomes to make and stably propagate heritable mutations in *WDY* (mutant stocks consist of $\hat{XX}$ *Y,WDY* females and $\hat{XY}$ *Y,WDY* males, see Methods, Figure S3 ^19^). Our crossing scheme also enabled us to identify and eliminate large chromosomal truncations that are common during genetic editing of the Y chromosome, likely due to its highly repetitive nature ^19^. To evaluate the phenotype of our mutants, we then removed the compound chromosomes by selective breeding to generate X Y, *WDY* mutant males. We confirmed each phenotype with three different *WDY* alleles, *F8*, *C104*, and *C3*, containing deletions of 547 bp, 545 bp and 443 bp, respectively (Figure S4, Table S4). All are large deletions close to the N-terminus that disrupt the reading frame and are therefore expected to be null; all three gave the same phenotype. We compared mutants to controls that account for the genetic background (Y^Tomato^) or crossing scheme (Y^C7^ and Y^G107^). In individual crosses to females from a wild-type strain (Canton S), control males produced progeny while *WDY* males were sterile (Table S5).

**Figure 1:**
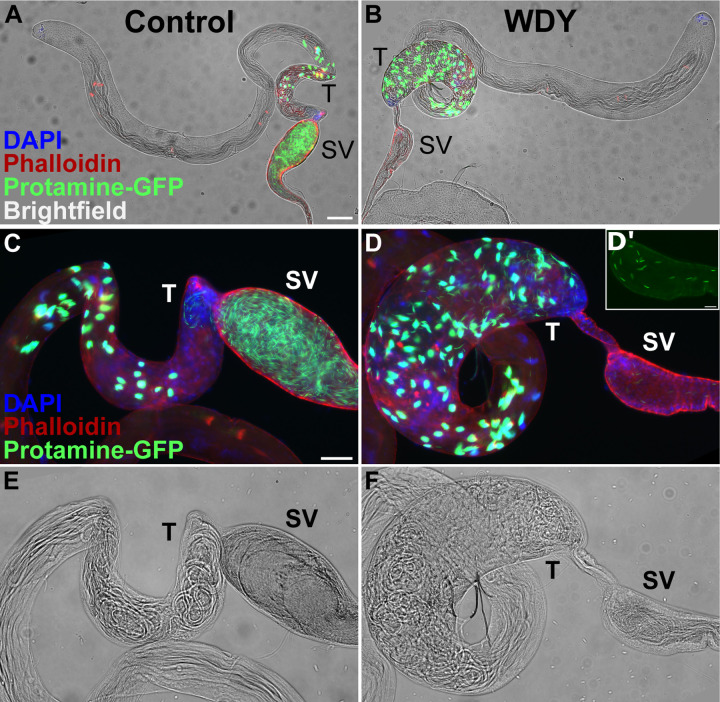
Spermatogenesis is backed up in *WDY* testes, but some mature sperm are found in the seminal vesicle Whole testes from control (A) and *WDY* mutant (B) males whose sperm were labelled with Protamine-GFP (green), Phalloidin (red) and DAPI (blue) overlaid on brightfield images. C-F Higher magnification view of posterior testes and seminal vesicle. Inset (D’) shows Protamine-labelled sperm in seminal vesicle of mutant. Testes (T) and seminal vesicle (SV) are marked. Bar denotes 100 µm for A-B, 50 µm for C-F, and 20 µm for D’.

To investigate the cause of this sterility, we first examined the distribution of sperm in the testes using Protamine-GFP ^22^, which labels sperm heads. Sperm of mutants in Y-linked fertility factors kl-2, kl-3,kl-5, ks-1, and ks-2 are eliminated before this time, during the individualization stage^19,23,24^. In contrast, we observed an accumulation of *WDY* mutant sperm in the posterior-most section of the testes, where individualized sperm accumulate while sperm coiling occurs, causing that region to bulge in the mutant (Figure 1). Sperm coiling is thought to function as a quality control step during which sperm with abnormal tails are eliminated by ingestion by the terminal epithelium ^25^. The accumulation of *WDY* sperm in the posterior testes may be due to their progression being stalled by this quality control mechanism or may indicate insufficient motility to exit the testes.

In the seminal vesicles there were fewer sperm in *WDY* mutants than controls (Figure 1C, D). Yet tails of sperm from both *WDY* and control males were observed to beat after we tore open the seminal vesicles (Movie M1,2). We observed no obvious differences in the movement of *WDY* versus control sperm. These observations match Kiefer’s conclusion that *kl-1* mutants were sterile but produced seemingly motile sperm ^17^. Our results that *WDY* mutations are sufficient to result in sterility, yet produce sperm that are motile, make it highly likely that *WDY* is the fertility factor known in the literature as *kl-1*.

### *WDY* mutant sperm are transferred to females, but do not enter the storage organs

We next tracked the movement of Protamine-labelled sperm in the RT of wild-type (Canton S) females 30 min after the start of mating (mASM). Sperm from both control and *WDY* mutant males were found in the female’s uterus (bursa) (Figure S5) and their tails were observed to beat when dissected out of the uterus (Movie M3,4). In both *WDY* and control genotypes, an open or folded conformation of the uterus correlated with the presence or absence of sperm, respectively, as expected ^26,27^. We conclude that motile *WDY* sperm are transferred to females and that *WDY* seminal fluid induces conformational changes in the uterus.

**Figure 2:**
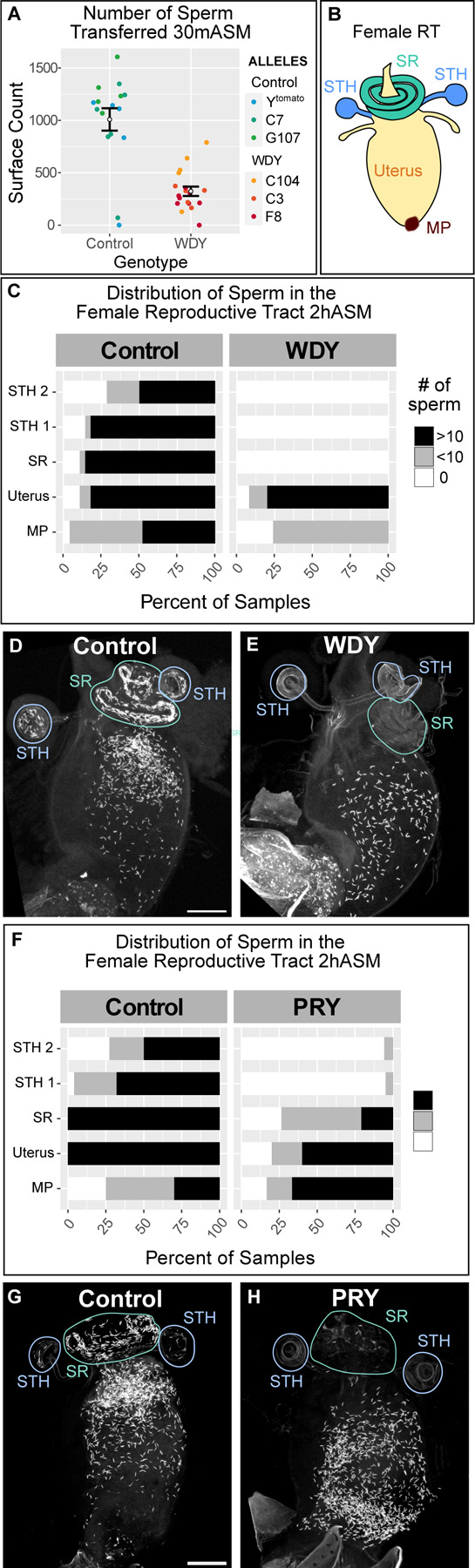
*WDY* and *PRY* mutant sperm fail to enter the storage organs in the female RT (A) Quantification of the number of sperm transferred to the female uterus 30 mASM to control or *WDY* mutant males. (B) Cartoon of the female RT indicating the mating plug (MP, brown), uterus (yellow), and the storage organs – the seminal receptacle (SR, green) and two spermathecae (STH, blue). (C) Quantification of the distribution of sperm within the female RT 2hASM. Representative images show Protamine-labelled sperm from Control (D) and *WDY* (E) mutant males within the female RT 2 hASM. (F) Quantification of the distribution of sperm within the female RT 2 hASM. Representative images show Protamine-labelled sperm from Control (G) and *PRY* (H) mutant males within the female RT 2 hASM. Bar denotes 100 µm.

We did, however, observe defects in the number of sperm transferred to, and the distribution of the sperm within, the female RT. *WDY* males transferred less than half as many sperm as control males as quantified at 30 mASM (Figure 2A, p<0.001). After mating, Drosophila sperm move rapidly from the uterus into either the primary storage organ, the seminal receptacle, or one of two long-term storage organs, the spermathecae ^28^ (Figure 2B). At 30 mASM most control samples contained some stored sperm, but no *WDY* sperm were found in the storage organs. At 2 hASM maximal numbers of sperm are stored in most control samples ^29^. Yet, again, no sperm from *WDY* mutants were seen in storage (Figure 2C-E). We also examined sperm in RTs of females left to mate overnight to see if a longer time or multiple mating may enable sperm to enter storage (Figure S5). Control samples all had stored sperm. *WDY* sperm were regularly observed in the uterus but never in any of the storage organs. We conclude that *WDY* mutant sperm are unable to enter the storage organs. In many animals, storage is required for sperm to become competent for fertilization ^30,31^, thus the lack of sperm storage might explain why *WDY* males are sterile.

### *WDY* mutant sperm in the male seminal vesicle and female uterus have decreased beat frequency

**Figure 3:**
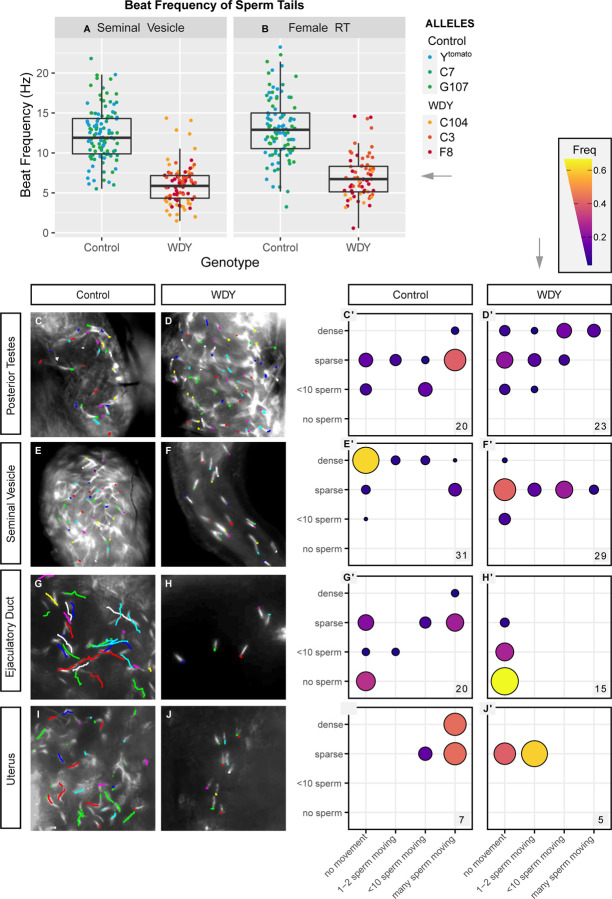
*WDY* mutant sperm have reduced beating frequency and do not swim in the female RT Quantification of tail beating frequency of *WDY* and control sperm dissected from seminal vesicles (A) and the female RT 30 mASM (B). Manual tracking of Protamine-GFP-labelled control (C,E,G,I) and *WDY* mutant (D,F,H,J) sperm heads over a 0.5 sec interval for representative videos of the posterior testes (C,D), seminal vesicle (E,F), ejaculatory duct (G,H), and uterus 1h ASM (I,J). (C’-J’) Corresponding quantification of the number of sperm heads and degree of movement observed from videos of each region of the RTs. The number of each type of organ that was scored is indicated.

Although *WDY* mutant sperm beat visibly in vitro (Movie M1–4), we wished to test whether subtle motility defects prevent them from being able to enter storage. We measured the beat frequency of sperm tails, by recording videos of control and *WDY* mutant sperm dissected directly from the male seminal vesicle or from the female uterus at 30 mASM (Methods, Movie M1–4, Figure 3A, B). In the seminal vesicle, the fastest *WDY* mutant sperm tails beat at an average of 6.0 Hz, whereas the fastest tail-beats by control sperm average 12.3 Hz (p<0.001). In the female uterus, the fastest *WDY* mutant sperm tails beat an average of 7.0 Hz, whereas the fastest control sperm tails beat at an average of 13.1 Hz (p<0.001). We conclude that *WDY* mutant sperm have a lower tail-beat frequency than wild type sperm in both the male and female RTs.

### *WDY* mutant sperm are unable to swim in the male ejaculatory duct and female uterus

We hypothesized that the lower beat frequency affects the ability of sperm to propel themselves. To test for defects in sperm swimming, sperm movement was assessed in videos by tracking the Protamine-labelled heads of control and *WDY* mutant sperm. In all regions the swimming speed of individual sperm varied, but there was an overriding regional pattern to the motility (Figure 3C-J). In mammals, sperm leaving the testes are immotile and must go undergo “epididymal maturation” in order to gain the ability to move progressively and to fertilize eggs ^32^. It was previously suggested, and often repeated, that, similarly, Drosophila sperm do not gain motility until they reach the seminal vesicle ^33^. We were surprised to see some individualized sperm heads slowly swimming within the posterior testes of most control samples (Figure 3C,C’, Movie M5). This suggests that in Drosophila sperm motility is normally initiated within the testes. *WDY* mutant sperm heads in this region also often moved around, suggesting that at least some mutant sperm develop motility (Figure 3D,D’, Movie M6).

Individual sperm heads generally ceased to move in the seminal vesicles of both control and *WDY* mutant flies, while flagella remained beating. However, mass movements occurred from contractions of the whole organ. It was unclear whether dense packing of sperm or some physical or chemical property of the seminal vesicle caused the immobilization of sperm heads while sperm tails continued to beat vigorously (Figure 3E,E’, Movie M7). This highlights that flagellar beating does not necessarily correlate with sperm swimming (i.e. moving through space). There were far fewer sperm in *WDY* mutant seminal vesicles, but the mutant sperm heads were predominantly immobilized, as in controls (Figure 3F,F’, Movie M8). We conclude that at least some *WDY* sperm develop the ability to swim in the testes and become immobilized in the seminal vesicle, as normal.

In contrast, a striking difference was seen in the ability of *WDY* sperm to swim beyond the seminal vesicle. In samples where sperm were found in the ejaculatory duct, control sperm heads were observed to move swiftly while *WDY* sperm heads appeared motionless (Figure 3G-H, G’-H’, Movie M9–10), and the same pattern was observed for sperm heads in the uterus 1 hASM. These sperm appear to be alive, as the tails continue to beat in place (Figure 3A,B). The lack of swimming *WDY* sperm in the uterus likely explains the inability of *WDY* sperm to enter the storage organs, though other defects may also exist and contribute ^34^. That *WDY* sperm in the posterior testes can swim suggests either (1) there is a subclass of *WDY* sperm that are capable of swimming but degenerate or (2) *WDY* mutant sperm are unable to navigate between different regions of the RTs.

### Significant hydrophobicity differences in putative calcium-binding residues coincide with *WDY*’s transition to Y-linkage in *Drosophila melanogaster*

**Figure 4:**
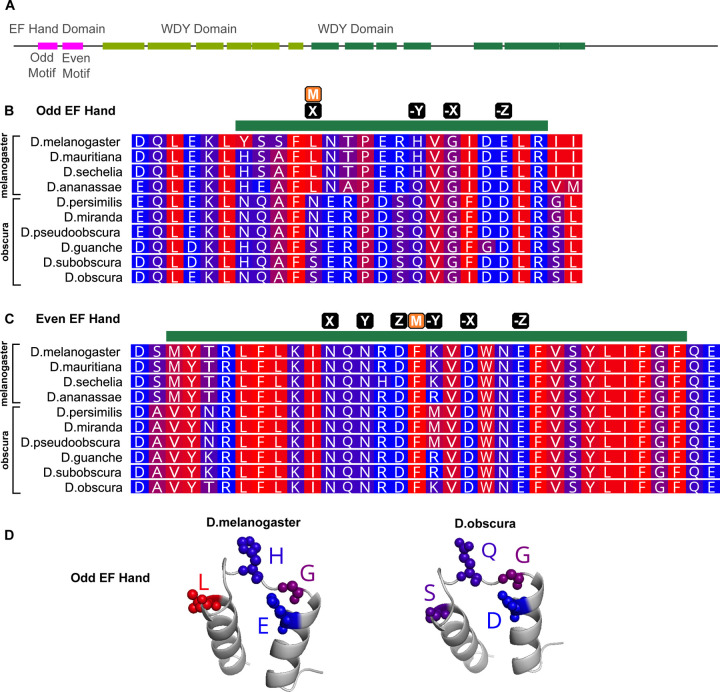
Difference in hydrophobicity in the EF Hand Domain in Y-linked orthologues of *WDY* in *Drosophila melanogaster* and its closest relatives (A) Domain structure of WDY (B,C) Protein alignment of melanogaster and obscura group species for the region with the Odd (Pseudo) (B) and Even (Canonical) (C) EF Hand motif. Green bar indicates the motif, M indicates the position of any mismatch between the *melanogaster* sequence and the consensus, and black boxes indicate putative calcium-binding residues (X,Y,Z,-X,-Y,-Z). (D) Predicted AlphaFold structure of Odd EF Hand domain with putative calcium binding residues labelled, generated in PyMOL. Blue-red scale indicates hydrophobicity (Red is hydrophobic, Blue is hydrophilic).

Calcium regulates sperm motility in many organisms, including humans ^39^ and Drosophila ^40–42^. WDY’s amino acid sequence contains a calcium-binding domain signature: an EF Hand (Interpro ^36^). Functional EF Hand domains contain a pair of motifs, each consisting of a loop flanked by alpha helices, that can bind Ca^2+^ ions. The specific characteristics of the loop affect calcium-binding affinity ^37^. We identified a putative pseudo EF hand motif followed by a canonical EF hand motif in WDY (Methods, Figure 4A-C). Known calcium-binding proteins (e.g. Calbindin D9K ^38^) also display this configuration. We also improved the annotation of two WD40 domains (Methods, Figure 4A), which typically mediate protein-protein interactions in protein complex assembly and/or signal transduction. Based on these findings, we speculate that *WDY* is necessary for sperm to recognize and adjust their motility based on differences in calcium in different regions of the RT.

We also compared sequences of the EF Hand domain between WDY orthologs in the melanogaster species group, where WDY is Y-linked, and the obscura species group, where WDY homologs are autosomal or X-linked ^4^. While there was relative conservation of the domain within each group (82.4% identical sites in each group), there were notable differences between the groups (63.2% identical sites overall), particularly in the loop residues of the Odd EF Hand motif (Figure 4B). The transition at position “X” is particularly compelling, since it involves a profound biochemical change in a conserved residue thought to directly bind calcium ^38^. The shift away from canonical residues in the melanogaster group could indicate a modulation of calcium binding, and thus significant functional evolution in the EF Hand domain, coinciding with Y-linkage.

### *PRY* is also required for efficient sperm storage

We previously generated and characterized mutants in another Y-linked gene, *PRY,* whose phenotype was consistent with abnormal sperm storage in females: mutants had low levels of fertility on the first day after mating but no fertility on subsequent days ^19^. Our finding that *WDY* affects sperm entry to the storage organs led us to wonder whether *PRY* affects a similar step. In contrast to *WDY*, *PRY* mutant sperm do swim in the female RT (Movie M13). However, the number of *PRY* sperm stored was significantly reduced compared to controls at 2 hASM (Figure 2F-H). *PRY* mutant sperm were frequently absent or reduced in the seminal receptacle, and rarely observed in the spermathecae. The number of stored *PRY* sperm was similar in RTs 2 hASM and 24 hASM (Figure S5) – defined times after a single, observed mating. However, significantly more sperm entered storage organs if males and females were housed together overnight (Figure S5). Seminal fluid proteins responsible for long term physiological effects of mating on females, including the inhibition of remating, can bind to sperm tails ^2,43–45^. Thus, lack of stored *PRY* sperm may lead to increased remating in these females.

Overall, we present functional evidence for a role for two Y-linked genes in sperm storage and demonstrate that *WDY* mutants have specific defects in sperm motility. Two broad themes emerge from this work. First, sperm may be unable to enter storage for different reasons – insufficient swimming speeds or an inability to navigate or gain entry into the storage organs. We demonstrate that sperm motility defects do manifest in an inability to enter storage. Female secretions are necessary to promote sperm storage^46^ so entry to sperm storage may be a hurdle imposed by females to ensure that only sperm with a certain level of motility/fitness are able to fertilize eggs. Higher remating rates upon lower sperm storage, as we appear to see with *PRY,* would then likely contribute to elimination of such sperm from the female RT through sperm competition.

Second, across species Y-linked genes appear to show ‘functional coherence’ ^6,23^. Even within the realm of male fertility, a disproportionate number of Y-linked genes seem to be singularly focused on aspects of sperm motility ^15,16^. Three axonemal dyneins were previously discovered on the Drosophila Y chromosome ^15,16^. However, since no sperm are produced upon genetic ablation of five out of the six fertility factor genes, the role of these genes in sperm motility has never been observed ^13,14,19,23^. We now add strength and stringency to the picture of functional coherence on the Drosophila Y chromosome. On the one hand being Y-linked allows sperm motility genes to escape the problems of countervailing selection in females (sexual conflict). However, being Y-linked bears the cost of not being able to recombine, which reduces the efficacy of natural selection (the Hill-Robertson effect). The fact that so many sperm motility genes are retained on the Y chromosome indicates a dynamic balance between these two opposing selective forces in different regions of the genome.

## Supplementary Material

Supplement 1
