# Supplementary material for: The Drosophila melanogaster Y-linked gene, WDY, is required for sperm to swim in the female reproductive tract
Source: bioRxiv. 2023 Feb 23:2023.02.02.526876. Originally published 2023 Feb 3. Preprint. [Version 2] doi: 10.1101/2023.02.02.526876 (PMC9915733; doi:10.1101/2023.02.02.526876)
Supplement: Supplement 1 [file NIHPP2023.02.02.526876v2-supplement-1.pdf]

bioRxiv preprint doi: https://doi.org/10.1101/2023.02.02.526876; this version posted February 23, 2023. The copyright holder for this preprint (which was not certified by peer review) is the author/funder, who has granted bioRxiv a license to display the preprint in perpetuity. It is made available under aCC-BY 4.0 International license.

SUPPLEMENTARY MATERIALS

Table S1: Drosophila Stocks

Full Genotype	Source	Notes
y[1] w[*] P{y[+t7.7]=nos-phiC31\int.NLS}X; PBac{y[+]-attP-9A}VK00027	BL #35569	phiC31/attP9A line used to make guide-expressing flies, from Rainbow Transgenics
y[1] w[*]; wg[Sp-1]/CyO; Dr[1]/TM3, Sb[1]	BL #59967	for balancing transformants
w[*]; TM3,Ser /TM6B, Tb	Wolfner Lab Stocks	for balancing transformants
w[1118]/TI{Disc\RFP[tdTom.3xP3]=attP}AByG	BL #78567	labelled Y chromosome
C(1;Y)3, In(1)FM7, v[Of]/C(1)M4, y[1]/0	BL #988	Source of compound X^X
C(1;Y)1, y[1]/0; ry[506]	BL #4408	Source of compound X^Y
w[*]; {U6:3-gRNA 3xWDY, w+}attP-9A / TM3	this study	transformant, expresses *WDY* and *ebony* guides ubiquitously
w Protamine-GFP, w+ ;; Bam-Gal4, UAS-Dicer	Chen and McKearin 2003, Manier et al 2010	Combined into one stock by Caroline Sartain
y[1] w[118] ; {nos-Cas9}attP2 / TM3	Kondo et al 2020	nos-Cas9 on III
yw ; {UAS-WDY RNAi}attP	VDRC_109045	WDY RNAi Line
yw ; {UAS-kl3 RNAi}attP	VDRC_109151	Kl-3 RNAi line
yw ; attP VIE-260B	VDRC_60100	RNAi Control Vie260
C(1;Y)1, y[1]/C(1)M4, y[1]/TI{Disc\RFP[tdTom.3xP3]=attP}AByG, WDY[F8]	this study	WDY F8 allele
C(1;Y)1, y[1]/C(1)M4, y[1]/TI{Disc\RFP[tdTom.3xP3]=attP}AByG,	this study	WDY C104 allele
C(1;Y)1, y[1]/C(1)M4, y[1]/TI{Disc\RFP[tdTom.3xP3]=attP}AByG, WDY[C3]	this study	WDY C3 allele
C(1;Y)1, y[1]/C(1)M4, y[1]/TI{Disc\RFP[tdTom.3xP3]=attP}AByG, [C7]	this study	Control C7 allele
C(1;Y)1, y[1]/C(1)M4, y[1]/TI{Disc\RFP[tdTom.3xP3]=attP}AByG, [G107]	this study	Control G107 allele

bioRxiv preprint doi: https://doi.org/10.1101/2023.02.02.526876; this version posted February 23, 2023. The copyright holder for this preprint (which was not certified by peer review) is the author/funder, who has granted bioRxiv a license to display the preprint in perpetuity. It is made available under aCC-BY 4.0 International license.

Table S2: Guide Sequences

Guide Name	Guide Sequence	PAM	Length	Reference
ebony	GCCACAATTGTCGATCGTCA	AGG	20	Kane et al. 2017
WDY 2.4n	AAGTATAGAGGTCGCTGCCC	TGG	20	
WDY 2.5n	ATAGTTTGGGATCCTTGGAC	AGG	20	
WDY 2.6n	CATAACATTGTGGTAACAGG	AGG	20	

Table S3: Primer Sequences

#	Name	Length	Sequence	Comments
**CRISPR plasmid construction primers**				
YH114	e_CP_1F	59	TTCCCGGCCGATGCAGCCACAATTGT CGATCGTCAGTTTAAGAGCTATGCTG	[pAC-U63-tgRNA-Rev tRNA Overhang] + [*ebony* guide] + [pMGC core primer]
YH115	WDY_CP_1R	37	CCTGTTACCACAATGTTATGTGCACCA GCCGGGAATC	[pMGC tRNA primer] + [WDY2.6n guide]
YH116	WDY_CP_2F	44	CATAACATTGTGGTAACAGGGTTTAA GAGCTATGCTGGAAACAG	[WDY2.6n guide] + [pMGC core primer]
YH117	WDY_CP_2R	37	GGGCAGCGACCTCTATACTTTGCACC AGCCGGGAATC	[pMGC tRNA primer] + [WDY2.4n guide]
YH118	WDY_CP_3F	44	AAGTATAGAGGTCGCTGCCCGTTTAA GAGCTATGCTGGAAACAG	[WDY2.4n guide] + [pMGC core primer]
YH119	WDY_CP_3R	57	TTCCAGCATAGCTCTTAAACGTCCAAG GATCCCAAACTATTGCACCAGCCGGG	[pMGC tRNA primer] + [WDY2.5n guide] + [pAC-U63-tgRNA-Rev Core
***WDY* genomic target site primers**				
ST20	WDY_3F	27	TGAGATGGTATCTTGCGTTTACTTTTC	Tm = 60°
ST21	WDY_3R	27	ACTTCTTGGTCTGGCATTATACTCATA	Tm = 60°
YH96	tgRNA_3F	20	GCCTCGAGTTAACGTTACGT	Tm = 58°, used to check plasmid and transgenic lines
YH97	tgRNA_2R	21	CGTCAACGGAAAACCATTGTC	Tm = 58.4°, used to check plasmid and transgenic lines

Table S4: Alleles Generated

Allele	Characteristics of the allele
Y_Tomato	unedited marked Y chromosome
Control_G107	went through crossing scheme but showed no changes at target site
Control_C7	went through crossing scheme but showed no changes at target site
WDY[F8]	547bp deletion between guides 2.4n-2.5n
WDY[C104]	545bp deletion between guides 2.4n-2.5n
WDY[C3]	443bp deletion between guides 2.6n-2.5n + 3bp change at guide 2.4n

bioRxiv preprint doi: https://doi.org/10.1101/2023.02.02.526876; this version posted February 23, 2023. The copyright holder for this preprint (which was not certified by peer review) is the author/funder, who has granted bioRxiv a license to display the preprint in perpetuity. It is made available under aCC-BY 4.0 International license.

Table S5: Sterility Test of *WDY*

Genotype	Allele	Fertile	Sterile
Control	tdTomato	18	0
Control	C7	17	0
Control	G107	17	0
WDY	F8	0	19
WDY	C104	0	19
WDY	C3	0	19
Control	RNAi	18	1
WDY	RNAi	5	15
kl-3	RNAi	0	10

Table S6: WDY Orthologue Sequences

Species	Source of Protein Sequence
D.melanogaster	NCBI : NP_001303588.1
D.simulans	Ching-Ho Chang, Lauren E Gregory, Kathleen E Gordon, Colin D Meiklejohn, Amanda M Larracuente (2022) Unique structure and positive selection promote the rapid divergence of Drosophila Y chromosomes eLife 11:e75795
D.mauritiana	
D.sechelia	
D.ananassae	GenBank: EU362855.1
D.persimilis	NCBI: XM_002020840.2
D.miranda	NCBI: XM_017296953.2
D.pseudoobscura	GenBank: DAA06444.1
D.guanche	NCBI: XM_034273953.1
D.subobscura	NCBI: XM_034814305.1
D.obscura	NCBI: XM_022365657.2

Figure S1: CRISPR target site on exon 2 of *WDY* had no identifiable duplicates.
Exon structure of *WDY* (introns not shown) with CRISPR target site marked. Purple arrows indicate primers, red triangles indicate guides. Additional copies of *WDY* regions on the Y chromosome are displayed as grey bars underneath the *WDY* schematic.

Figure S2: Position effect variegation observed in some lines after CRISPR editing at *WDY*.
Adult eyes of females with compound-X containing the position effect variegation marker, w^{M4}, and no Y chromosome (A), an unedited Y chromosome (B), or CRISPR-edited Y chromosomes G17 (C) or C12 (D).

Figure S3: Crossing scheme for inducing CRISPR, then balancing the edited Y chromosome using compound-X and attached X-Y chromosomes.

Figure S4: Sequencing results in target region for *WDY* and control alleles used in this study.
Guide sites are marked with red boxes. Nucleotide changes are highlighted. Gaps represent deletions.

bioRxiv preprint doi: https://doi.org/10.1101/2023.02.02.526876; this version posted February 23, 2023. The copyright holder for this preprint (which was not certified by peer review) is the author/funder, who has granted bioRxiv a license to display the preprint in perpetuity. It is made available under aCC-BY 4.0 International license.

Figure S5: Distribution of *WDY* and *PRY* mutant sperm in the female reproductive tract after mating. (A) Quantification of *WDY* mutant sperm's distribution in the female reproductive tract 30 mASM. (B) Quantification of *WDY* mutant sperm's distribution in the female reproductive tract after overnight mating. (C) Quantification of *PRY* mutant sperm's distribution in the female reproductive tract 24 hASM. (D) Quantification of *PRY* mutant sperm's distribution in the female reproductive tract after overnight mating.

Figure S1: CRISPR target site on exon 2 of WDY had no identifiable duplicates

bioRxiv preprint doi: https://doi.org/10.1101/2023.02.02.526876; this version posted February 23, 2023. The copyright holder for this preprint (which was not certified by peer review) is the author/funder, who has granted bioRxiv a license to display the preprint in perpetuity. It is made available under aCC-BY 4.0 International license.

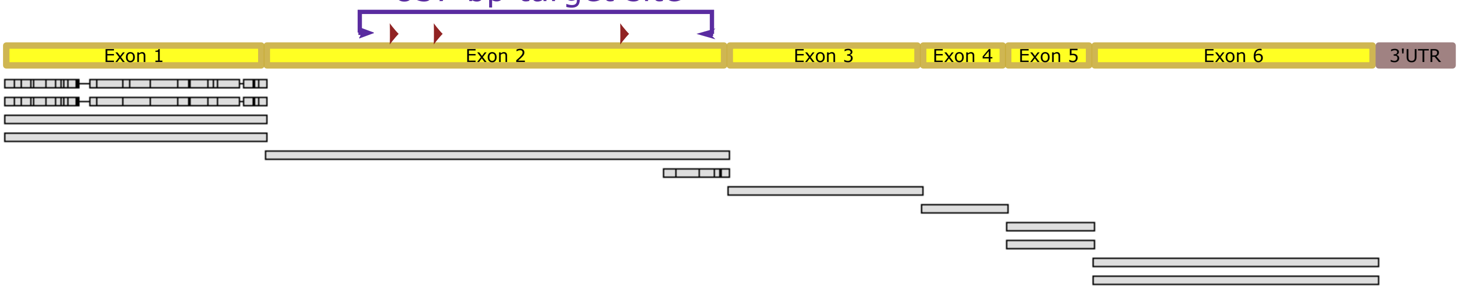

Figure S2: Position effect variegation observed in some lines after CRISPR editing at *WDY*

bioRxiv preprint doi: https://doi.org/10.1101/2023.02.02.526876; this version posted February 23, 2023. The copyright holder for this preprint (which was not certified by peer review) is the author/funder, who has granted bioRxiv a license to display the preprint in perpetuity. It is made available under aCC-BY 4.0 International license.

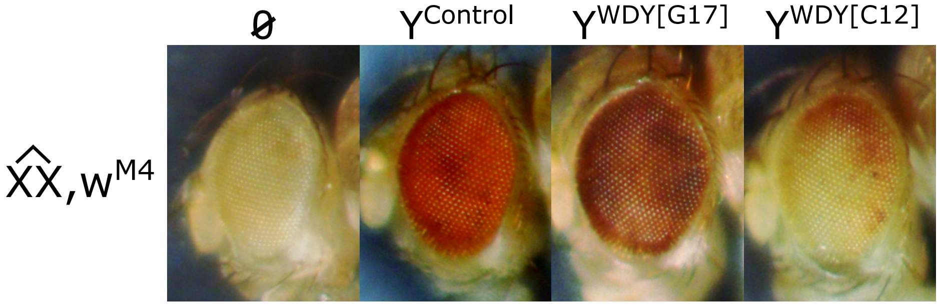

Figure S3: Crossing scheme for creating stable *WDY* mutants after CRISPR

bioRxiv preprint doi: https://doi.org/10.1101/2023.02.02.526876; this version posted February 23, 2023. The copyright holder for this preprint (which was not certified by peer review) is the author/funder, who has granted bioRxiv a license to display the preprint in perpetuity. It is made available under aCC-BY 4.0 International license.

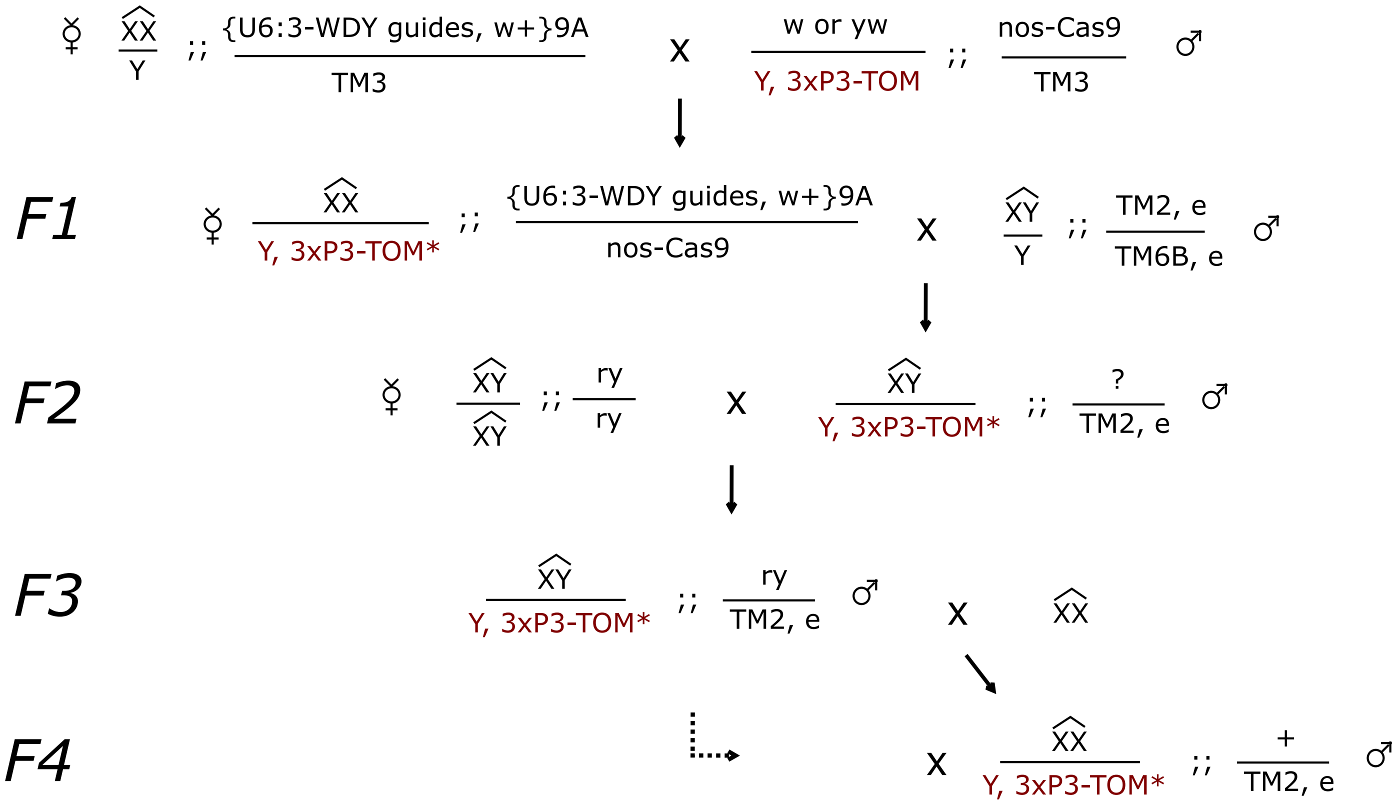

Figure S4: Molecular characterization of *WDY* alleles

bioRxiv preprint doi: https://doi.org/10.1101/2023.02.02.526876; this version posted February 23, 2023. The copyright holder for this preprint (which was not certified by peer review) is the author/funder, who has granted bioRxiv a license to display the preprint in perpetuity. It is made available under aCC-BY 4.0 International license.

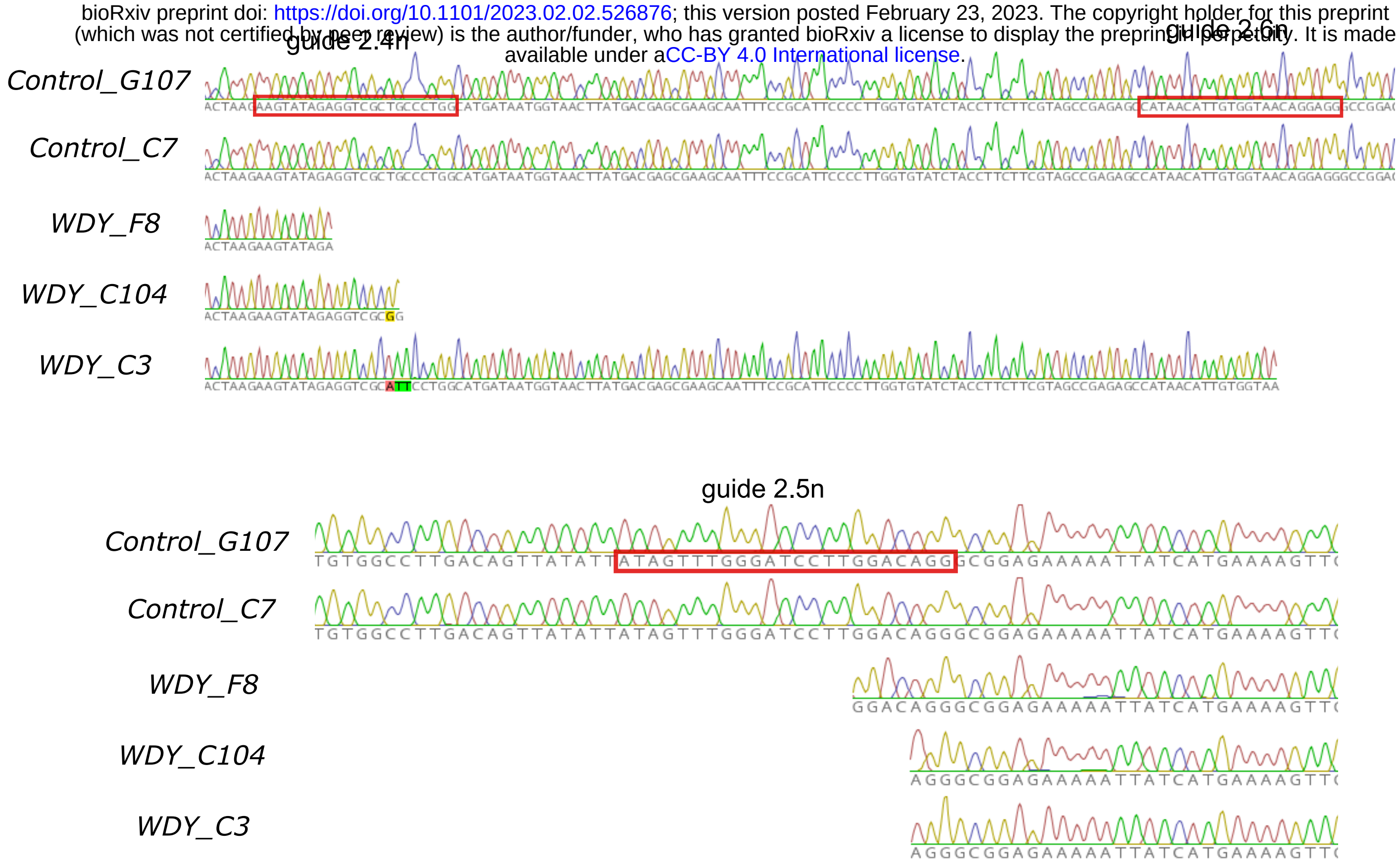

Figure S5: Distribution of *WDY* and *PRY* mutant sperm in the female reproductive tract after mating

bioRxiv preprint doi: https://doi.org/10.1101/2023.02.02.526876; this version posted February 23, 2023. The copyright holder for this preprint (which was not certified by peer review) is the author/funder, who has granted bioRxiv a license to display the preprint in perpetuity. It is made available under aCC-BY 4.0 International license.

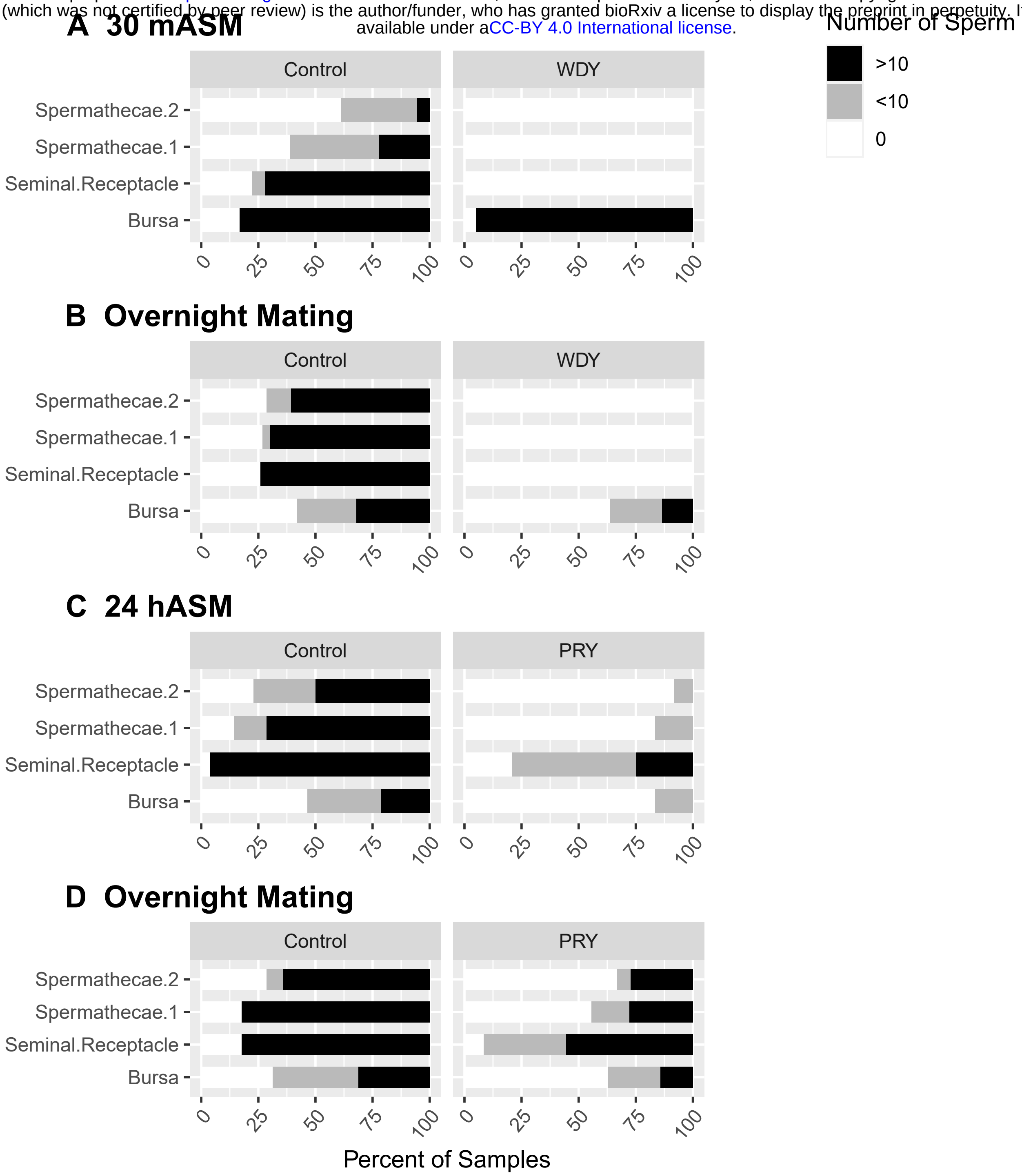

bioRxiv preprint doi: https://doi.org/10.1101/2023.02.02.526876; this version posted February 23, 2023. The copyright holder for this preprint (which was not certified by peer review) is the author/funder, who has granted bioRxiv a license to display the preprint in perpetuity. It is made available under aCC-BY 4.0 International license.

METHODS

Drosophila Stocks and Husbandry

Flies were reared on a cornmeal-agar-sucrose medium (recipe available at https://cornellfly.wordpress.com/s-food/) at 25°, with a 12 hr light-dark cycle. The stocks used in this study are described in Table S1.

Generation of a *WDY* Mutant with CRISPR

Three 20 base pair guide RNAs were designed to target exon 2 of *WDY*, a region of the gene with no known duplications (Figure S1) [1]. We also targeted *ebony*, a visible Co-CRISPR marker [2]. Guide sequences were incorporated into pAC-U63-tgRNA-Rev (Addgene, Plasmid #112811) which is analogous to the "tgFE" construct from [3]. This was done by appending guide RNA sequences to tracrRNA core and tRNA sequences from pMGC (Addgene, Plasmid #112812) through tailed primers (Table S3) to create inserts that were then inserted by Gibson Assembly into a *SapI*-digested pAC-U63-tgRNA-Rev (Addgene, Plasmid #112811). The plasmid backbone contained attB, and we used Phi-C31 to integrate it into an attP-9A site on chromosome 3R. The construct was injected into *yw nanos-phiC31; PBac{y+-attP-9A}VK00027* by Rainbow Transgenic Flies Inc. Transformants were identified by eye color from a *mini-white+* marker. Transformants express the four guides ubiquitously under the U6:3 promoter as a single polycistronic transcript that is processed by the endogenous cellular tRNA processing machinery (RNase P and Z) to release the individual mature gRNAs and interspersed tRNAs. The transformants were balanced and inserts were confirmed by PCR and sequencing. A few of the transformants had light-red eyes, but we only used those with dark-red eyes.

Different combinations of transformants and germline Cas-9 drivers were tested for editing efficiency (data not shown). Males containing *vasa-Cas9* and our guide RNAs were sterile, while females showed a 6.2% CRISPR efficiency, based on the generation of *ebony* mutants. In contrast, F1 males from crosses with *nanos-Cas9* drivers on chromosomes 2 and 3 produced progeny. F2 progeny (from male and female crosses combined) showed that these two lines had editing efficiencies of 2.2% and 2.6%, respectively. Our observation of higher efficiency and sterility from *vasa-Cas9* is consistent with the earlier [4] and higher somatic [5,6] protein expression of Vasa versus Nanos, as well as the RNAi phenotype of *WDY* [7]. We proceeded to make stable mutants by crossing transformant #1 to *nanos-Cas9* in a compound chromosome background.

Our crossing scheme for creating *WDY* alleles is shown in Figure S3. We combined the *nanos-Cas9* driver on chromosome 3 with a Y chromosome marked with *3xP3-tdTomato* [8]. We also combined the guide-expressing insert on 3R with a compound X ($\widehat{XX}$; *C(1)M4, y[1]*). CRISPR editing occurred in F1 females ($\widehat{XX}Y^{3XP3\text{-}tdTomato}$) that carried the marked Y chromosome. By crossing to a compound X-Y ($\widehat{XY}$, *C(1;Y)1, y[1]*) we were able to establish balanced lines from 55 *ebony* and 5 non-ebony F2 flies. Males were of genotype $\widehat{XY}$ $Y^{3XP3\text{-}tdTomato}$ and were fertile regardless of CRISPR-mediated edits of the free Y. We screened these lines for visible deletions in the *WDY* target site – first by gel, then by sequencing. Alleles derived from our crossing scheme are listed in Table S4 and described in Figure S4. They are maintained as stable lines with $\widehat{XX}Y$ females and $\widehat{XY}Y$ males; the free Y chromosome is edited.

bioRxiv preprint doi: https://doi.org/10.1101/2023.02.02.526876; this version posted February 23, 2023. The copyright holder for this preprint (which was not certified by peer review) is the author/funder, who has granted bioRxiv a license to display the preprint in perpetuity. It is made available under aCC-BY 4.0 International license.

In several of our lines, we saw varying, intermediate degrees of position effect variegation (PEV) (Figure S2). This corresponded with either failed amplification at the target site in *WDY* or the presence of several bands of unexpected size. Based on our previous results when editing *FDY* with CRISPR[7], we hypothesized that these mutations were large deletions in the Y chromosome, and thus did not phenotype these mutants for sperm or fertility characteristics. C(1)M4 contains *white*[*mottled-4*], a PEV marker that is highly sensitive to Y-chromosome dosage. $\widehat{XX}$ females with C(1)M4 have mostly white eyes, while $\widehat{XX}$ females have an almost entirely red eye. We previously showed that lines with visibly altered PEV lacked large sections of the Y chromosome[7]. Such deletions may be caused by the presence of uncharacterized copies of the target region present in unassembled regions of the Y chromosome.

Sterility, Mating, and Sperm Storage

Crosses and experiments were done with flies 2-5 days after eclosion (dAE). To test for sterility, we crossed individual XY males to 4 Canton S virgin females in a food containing vial with wet yeast. Adults were transferred to a new vial after one week. Crosses were scored for the presence of progeny. 15-20 crosses were tested per line. For experiments that required timing from the start of mating, one Canton S virgin female was mated to three males of a given genotype and flies were observed. Once mating began the time was noted. Females were analyzed or flash frozen in liquid nitrogen for 30 minutes, 2 hours, or 24 hours after the start of mating (30 mASM, 2 hASM, 24 hASM). Reproductive tracts were dissected from frozen females in PBS, fixed in 4% paraformaldehyde, and mounted in Vectashield with DAPI. Samples were imaged on an Echo Revolve microscope or a Leica DMRE confocal microscope.

Sperm Counting with Imaris software

To quantify sperm transferred, female reproductive tracts 30mASM were imaged on a Leica DMRE confocal using standardized settings. Two µm Z-stacks through each sample were collected. Using Imaris 9.8.0 software (RRID:SCR_007370), first the female reproductive tract was extracted in each image by manually drawing a contour surface. The mating plug and cuticle were specifically excluded due to their high autofluorescence [9] Second, protamine-labelled sperm heads were automatically detected using the "Surfaces" function (smoothing and background elimination enabled, 2.0 µm surface grain size, 1 µm diameter of largest sphere, 2.747-13.048 manual threshold, >15 quality, <0.8 sphericity). Counts of transferred sperm from control and *WDY* males were statistically compared using a Student's t-test in R software.

Sperm-tail Beat Frequency Analysis

Tail-beat frequency was measured for sperm dissected from the reproductive tracts of males 2-5dAE or females 2-5 dAE and 30 mASM into PBS. Sperm were released into a 15 ul drop of PBS on a glass slide by tearing the male seminal vesicle or female uterus. Sperm were observed under brightfield optics with an Olympus BX51WDI microscope and a 50x LMPLFLN objective. Eight second raw movie clips at 1280x720 resolution and 60 frames per second were captured from 4-6 different regions around the sperm mass using a Canon EOS Rebel T6 camera. Dissected sperm masses all contain sperm tails beating at a range

bioRxiv preprint doi: https://doi.org/10.1101/2023.02.02.526876; this version posted February 23, 2023. The copyright holder for this preprint (which was not certified by peer review) is the author/funder, who has granted bioRxiv a license to display the preprint in perpetuity. It is made available under aCC-BY 4.0 International license.

of frequencies - we specifically quantified the beat frequency of the 1-2 fastest-beating sperm tails from each clip.

To measure the sperm tail-beating frequency, video clips were imported to FIJI (RRID:SCR_002285) using the ffmpeg plug-in. From each clip, we measured beat frequencies of the 1-2 fastest-beating sperm, limited to tails that were not overlapping or entangled with other sperm tails. A selection line was drawn across an isolated section of sperm tail. A 1-pixel "Multi Kymograph" was generated which shows pixel intensities across the selection line on the X-axis for each frame along the Y-axis. The beating of the sperm tail appears as a traveling wave form. The number of beats and the number of frames were counted for the region where the sperm tail remained in focus and isolated from other tails. Beat frequency was then calculated as: Hz = (# beats X 60 fps) / # frames. Ten measurements were made per individual fly. Approximately one-third of samples were scored blind, and statistical analysis indicated consistent results whether samples were scored blind or not.

Sperm tail-beat frequencies were measured from a minimum of three individuals of each allele. Using the lme4 package in R, linear mixed models were fitted to the data, incorporating the individual as a random effect and experimental batch and the experimenter who measured beat frequency as fixed effects. We then ran a Likelihood-Ratio test to compare the model with and without "Genotype" as a fixed effect.

Sperm Swimming Analysis

Videos of sperm swimming were acquired from either male reproductive tracts or female reproductive tracts 1 hASM. Tracts were dissected and mounted in 15 µL PBS. Spacers (2 layers of double-stick tape) were used to avoid compression of the tissue by the coverslip. Fluorescent sperm heads were recorded through screen recording of the preview window on an Echo Revolve. We used ffmpeg (RRID:SCR_016075) to convert videos to constant frame rate of 60 fps and .mov format. Videos were then imported into FIJI (RRID:SCR_002285) using the ffmpeg plugin. We manually tracked sperm heads across 60 frames using the "Manual Tracking" plugin in FIJI. The tracking shown in Figure 3 represent movement across 30 frames.

WDY Annotation and Sequence Comparisons

EF hand motifs were identified by searching (using Geneious software, RRID:SCR_010519) for the canonical and pseudo PROSITE motif consensus sequences defined in [[10]] and allowing for a maximum 1 base pair mismatch. Because the pseudo-EF hand motif contains a variable size region, there were two potential start locations in the sequence – residue 44 or 47. However, the Alphafold prediction showed residues that should form the loop region would instead form part of the alpha helix in the motif beginning at residue 44. We therefore favored the motif beginning at residue 47. Locations of the calcium binding residues were determined based on the consensus sequence logograms in [[10]].

Three WD40 domains [11] were originally identified in the protein sequence based on homology. Flybase reported a handful of WD40 repeats (2 for Pfam and 8 for SMART) were identified. 4 to 16 of these repeat domains may together form a circular beta propeller structure called a WD40 domain [12–14];

bioRxiv preprint doi: https://doi.org/10.1101/2023.02.02.526876; this version posted February 23, 2023. The copyright holder for this preprint (which was not certified by peer review) is the author/funder, who has granted bioRxiv a license to display the preprint in perpetuity. It is made available under aCC-BY 4.0 International license.

however, insufficient WD40 repeats were identified in WDY to predict the presence of a WD40 domain. We used the a structural prediction of *D. melanogaster* WDY by Alphafold (PDB B4F7L9) [15] to identify the locations of the characteristic β-propeller, consisting of 4 antiparallel sheets [13]. WDY is predicted to form two WD40 domains - one with 6 WD40 repeats and one with 7 WD40 repeats.

WDY ortholog sequences were obtained as described in Table S6. The proteins were aligned in Geneious using a BLOSUM cost matrix with a gap open cost of 10 and a gap extend cost of 0.1.

REFERENCES

1. Chang, C.-H. & Larracuente, A. M. Heterochromatin-Enriched Assemblies Reveal the Sequence and Organization of the Drosophila melanogaster Y Chromosome. *Genetics* **211**, 333–348 (2019).
2. Kane, N. S., Vora, M., Varre, K. J. & Padgett, R. W. Efficient Screening of CRISPR/Cas9-Induced Events in Drosophila Using a Co-CRISPR Strategy. *G3 (Bethesda)* **7**, 87–93 (2016).
3. Poe, A. R. *et al.* Robust CRISPR/Cas9-Mediated Tissue-Specific Mutagenesis Reveals Gene Redundancy and Perdurance in Drosophila. *Genetics* **211**, 459–472 (2019).
4. Siddall, N. A. & Hime, G. R. A Drosophila toolkit for defining gene function in spermatogenesis. *Reproduction* **153**, R121–R132 (2017).
5. Champer, J. *et al.* Reducing resistance allele formation in CRISPR gene drive. *Proc. Natl. Acad. Sci. U.S.A.* **115**, 5522–5527 (2018).
6. Renault, A. D. vasa is expressed in somatic cells of the embryonic gonad in a sex-specific manner in Drosophila melanogaster. *Biol Open* **1**, 1043–1048 (2012).
7. Hafezi, Y., Sruba, S. R., Tarrash, S. R., Wolfner, M. F. & Clark, A. G. Dissecting Fertility Functions of Drosophila Y Chromosome Genes with CRISPR. *Genetics* **214**, 977–990 (2020).
8. Buchman, A. & Akbari, O. S. Site-specific transgenesis of the Drosophila melanogaster Y-chromosome using CRISPR/Cas9. *Insect Mol Biol* **28**, 65–73 (2019).
9. Lung, O. & Wolfner, M. F. Identification and characterization of the major Drosophila melanogaster mating plug protein. *Insect Biochemistry and Molecular Biology* **31**, 543–551 (2001).
10. Zhou, Y. *et al.* Prediction of EF-hand calcium-binding proteins and analysis of bacterial EF-hand proteins. *Proteins: Structure, Function, and Bioinformatics* **65**, 643–655 (2006).
11. Vibranovski, M. D., Koerich, L. B. & Carvalho, A. B. Two new Y-linked genes in Drosophila melanogaster. *Genetics* **179**, 2325–2327 (2008).
12. Li, D. & Roberts, R. Human Genome and Diseases:¶WD-repeat proteins: structure characteristics, biological function, and their involvement in human diseases. *CMLS, Cell. Mol. Life Sci.* **58**, 2085–2097 (2001).
13. Jain, B. P. & Pandey, S. WD40 Repeat Proteins: Signalling Scaffold with Diverse Functions. *Protein J* **37**, 391–406 (2018).
14. Smith, T. F., Gaitatzes, C., Saxena, K. & Neer, E. J. The WD repeat: a common architecture for diverse functions. *Trends in Biochemical Sciences* **24**, 181–185 (1999).

bioRxiv preprint doi: https://doi.org/10.1101/2023.02.02.526876; this version posted February 23, 2023. The copyright holder for this preprint (which was not certified by peer review) is the author/funder, who has granted bioRxiv a license to display the preprint in perpetuity. It is made available under aCC-BY 4.0 International license.

15. Jumper, J. *et al.* Highly accurate protein structure prediction with AlphaFold. *Nature* **596**, 583–589 (2021).
