# Supplementary material for: The Drosophila melanogaster Y-linked gene, WDY, is required for sperm to swim in the female reproductive tract
Source: bioRxiv. 2023 Feb 3:2023.02.02.526876. Preprint. [Version 1] doi: 10.1101/2023.02.02.526876 (PMC9915733; doi:10.1101/2023.02.02.526876)

SUPPLEMENTARY MATERIALS

Table S1: Drosophila Stocks

Full Genotype	Source	Notes
y[1] w[*] P{y[+t7.7]=nos-phiC31\int.NLS}X; PBac{y[+]-attP-9A}VK00027	BL #35569	phiC31/attP9A line used to make guide-expressing flies, from Rainbow Transgenics
y[1] w[*]; wg[Sp-1]/CyO; Dr[1]/TM3, Sb[1]	BL #59967	for balancing transformants
w[*]; TM3,Ser /TM6B, Tb	Wolfner Lab Stocks	for balancing transformants
w[1118]/TI{Disc\RFP[tdTom.3xP3]=attP}AByG	BL #78567	labelled Y chromosome
C(1;Y)3, In(1)FM7, v[Of]/C(1)M4, y[1]/0	BL #988	Source of compound X^X
C(1;Y)1, y[1]/0; ry[506]	BL #4408	Source of compound X^Y
w[*]; {U6:3-gRNA 3xWDY, w+}attP-9A / TM3	this study	transformant, expresses *WDY* and *ebony* guides ubiquitously
w Protamine-GFP, w+ ;; Bam-Gal4, UAS-Dicer	Chen and McKearin 2003, Manier et al 2010	Combined into one stock by Caroline Sartain
y[1] w[118] ; {nos-Cas9}attP2 / TM3	Kondo et al 2020	nos-Cas9 on III
yw ; {UAS-WDY RNAi}attP	VDRC_109045	WDY RNAi Line
yw ; {UAS-kl3 RNAi}attP	VDRC_109151	Kl-3 RNAi line
yw ; attP VIE-260B	VDRC_60100	RNAi Control Vie260
C(1;Y)1, y[1]/C(1)M4, y[1]/TI{Disc\RFP[tdTom.3xP3]=attP}AByG, WDY[F8]	this study	WDY F8 allele
C(1;Y)1, y[1]/C(1)M4, y[1]/TI{Disc\RFP[tdTom.3xP3]=attP}AByG,	this study	WDY C104 allele
C(1;Y)1, y[1]/C(1)M4, y[1]/TI{Disc\RFP[tdTom.3xP3]=attP}AByG, WDY[C3]	this study	WDY C3 allele
C(1;Y)1, y[1]/C(1)M4, y[1]/TI{Disc\RFP[tdTom.3xP3]=attP}AByG, [C7]	this study	Control C7 allele
C(1;Y)1, y[1]/C(1)M4, y[1]/TI{Disc\RFP[tdTom.3xP3]=attP}AByG, [G107]	this study	Control G107 allele

Table S2: Guide Sequences

Guide Name	Guide Sequence	PAM	Length	Reference
ebony	GCCACAATTGTCGATCGTCA	AGG	20	Kane et al. 2017
WDY 2.4n	AAGTATAGAGGTCGCTGCCC	TGG	20	
WDY 2.5n	ATAGTTTGGGATCCTTGGAC	AGG	20	
WDY 2.6n	CATAACATTGTGGTAACAGG	AGG	20	

Table S3: Primer Sequences

#	Name	Length	Sequence	Comments
**CRISPR plasmid construction primers**				
YH114	e_CP_1F	59	TTCCCGGCCGATGCAGCCACAATTGT CGATCGTCAGTTTAAGAGCTATGCTG	[pAC-U63-tgRNA-Rev tRNA Overhang] + [*ebony* guide] + [pMGC core primer]
YH115	WDY_CP_1R	37	CCTGTTACCACAATGTTATGTGCACCA GCCGGGAATC	[pMGC tRNA primer] + [WDY2.6n guide]
YH116	WDY_CP_2F	44	CATAACATTGTGGTAACAGGGTTTAA GAGCTATGCTGGAAACAG	[WDY2.6n guide] + [pMGC core primer]
YH117	WDY_CP_2R	37	GGGCAGCGACCTCTATACTTTGCACC AGCCGGGAATC	[pMGC tRNA primer] + [WDY2.4n guide]
YH118	WDY_CP_3F	44	AAGTATAGAGGTCGCTGCCCGTTTAA GAGCTATGCTGGAAACAG	[WDY2.4n guide] + [pMGC core primer]
YH119	WDY_CP_3R	57	TTCCAGCATAGCTCTTAAACGTCCAAG GATCCCAAACTATTGCACCAGCCGGG	[pMGC tRNA primer] + [WDY2.5n guide] + [pAC-U63-tgRNA-Rev Core
***WDY* genomic target site primers**				
ST20	WDY_3F	27	TGAGATGGTATCTTGCGTTTACTTTTC	Tm = 60°
ST21	WDY_3R	27	ACTTCTTGGTCTGGCATTATACTCATA	Tm = 60°
YH96	tgRNA_3F	20	GCCTCGAGTTAACGTTACGT	Tm = 58°, used to check plasmid and transgenic lines
YH97	tgRNA_2R	21	CGTCAACGGAAAACCATTGTC	Tm = 58.4°, used to check plasmid and transgenic lines

Table S4: Alleles Generated

Allele	Characteristics of the allele
Y_Tomato	unedited marked Y chromosome
Control_G107	went through crossing scheme but showed no changes at target site
Control_C7	went through crossing scheme but showed no changes at target site
WDY[F8]	547bp deletion between guides 2.4n-2.5n
WDY[C104]	545bp deletion between guides 2.4n-2.5n
WDY[C3]	443bp deletion between guides 2.6n-2.5n + 3bp change at guide 2.4n

Table S5: Sterility Test of *WDY*

Genotype	Allele	Fertile	Sterile
Control	tdTomato	18	0
Control	C7	17	0
Control	G107	17	0
WDY	F8	0	19
WDY	C104	0	19
WDY	C3	0	19
Control	RNAi	18	1
WDY	RNAi	5	15
kl-3	RNAi	0	10

Table S6: WDY Orthologue Sequences

Species	Source of Protein Sequence
D.melanogaster	NCBI : NP_001303588.1
D.simulans	Ching-Ho Chang, Lauren E Gregory, Kathleen E Gordon, Colin D Meiklejohn, Amanda M Larracuente (2022) Unique structure and positive selection promote the rapid divergence of Drosophila Y chromosomes eLife 11:e75795
D.mauritiana	
D.sechelia	
D.ananassae	GenBank: EU362855.1
D.persimilis	NCBI: XM_002020840.2
D.miranda	NCBI: XM_017296953.2
D.pseudoobscura	GenBank: DAA06444.1
D.guanche	NCBI: XM_034273953.1
D.subobscura	NCBI: XM_034814305.1
D.obscura	NCBI: XM_022365657.2

Figure S1: CRISPR target site on exon 2 of *WDY* had no identifiable duplicates.
Exon structure of *WDY* (introns not shown) with CRISPR target site marked. Purple arrows indicate primers, red triangles indicate guides. Additional copies of *WDY* regions on the Y chromosome are displayed as grey bars underneath the *WDY* schematic.

Figure S2: Position effect variegation observed in some lines after CRISPR editing at *WDY*.
Adult eyes of females with compound-X containing the position effect variegation marker, w^{M4}, and no Y chromosome (A), an unedited Y chromosome (B), or CRISPR-edited Y chromosomes G17 (C) or C12 (D).

Figure S3: Crossing scheme for inducing CRISPR, then balancing the edited Y chromosome using compound-X and attached X-Y chromosomes.

Figure S4: Sequencing results in target region for *WDY* and control alleles used in this study.
Guide sites are marked with red boxes. Nucleotide changes are highlighted. Gaps represent deletions.

Figure S5: Distribution of *WDY* and *PRY* mutant sperm in the female reproductive tract after mating. (A) Quantification of *WDY* mutant sperm's distribution in the female reproductive tract 30 mASM. (B) Quantification of *WDY* mutant sperm's distribution in the female reproductive tract after overnight mating. (C) Quantification of *PRY* mutant sperm's distribution in the female reproductive tract 24 hASM. (D) Quantification of *PRY* mutant sperm's distribution in the female reproductive tract after overnight mating.

Figure S1: CRISPR target site on exon 2 of WDY had no identifiable duplicates

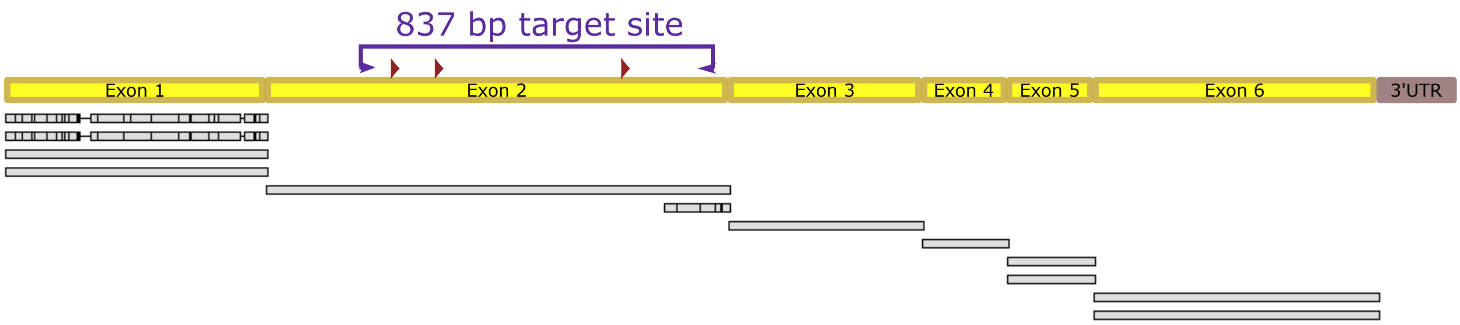

Figure S2: Position effect variegation observed in some lines after CRISPR editing at *WDY*

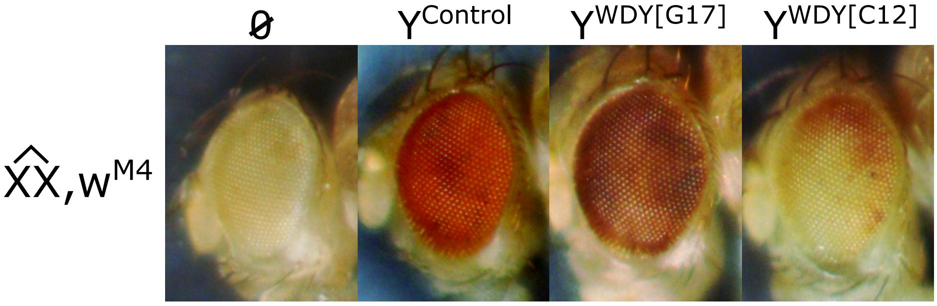

Figure S3: Crossing scheme for creating stable *WDY* mutants after CRISPR

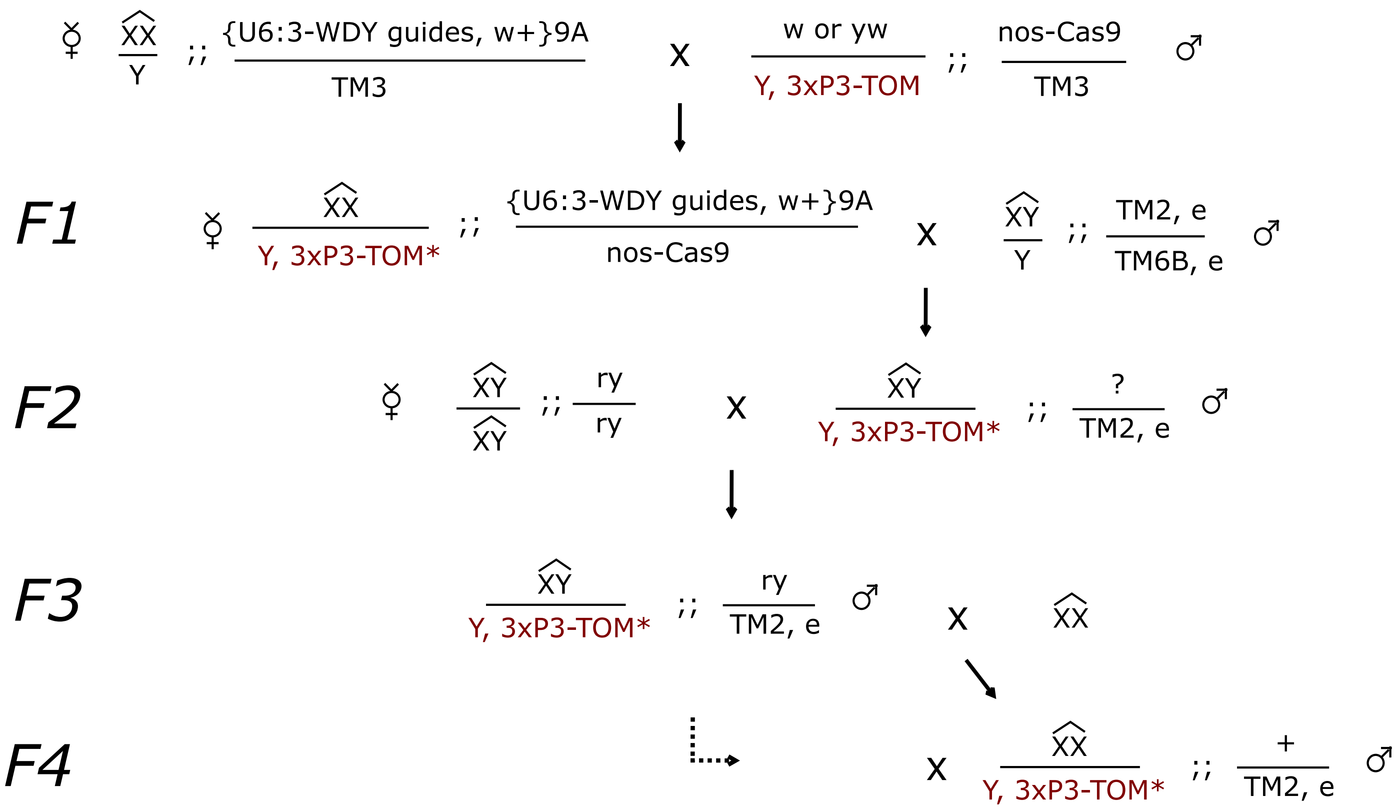

Figure S4: Molecular characterization of *WDY* alleles

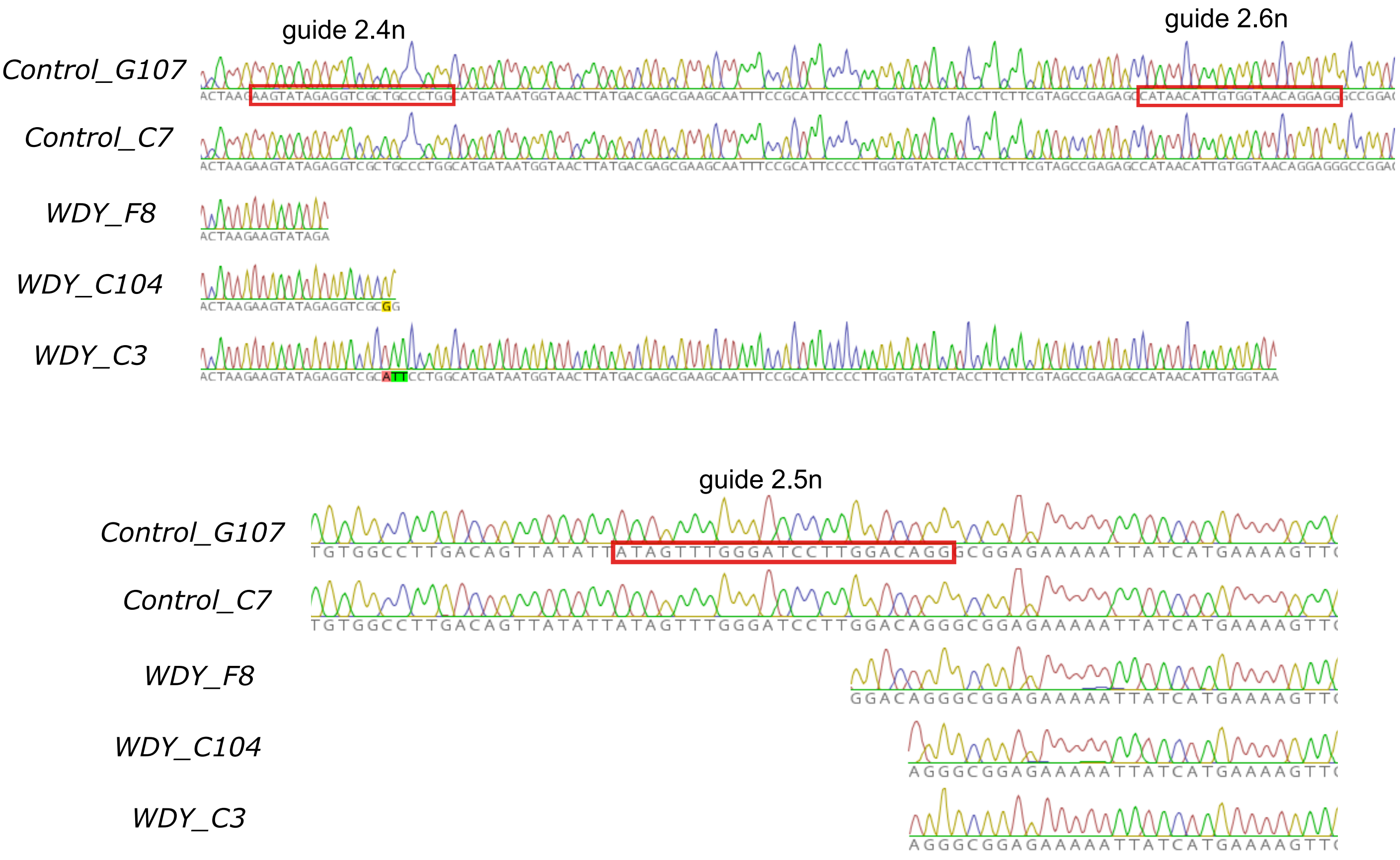

Figure S5: Distribution of *WDY* and *PRY* mutant sperm in the female reproductive tract after mating

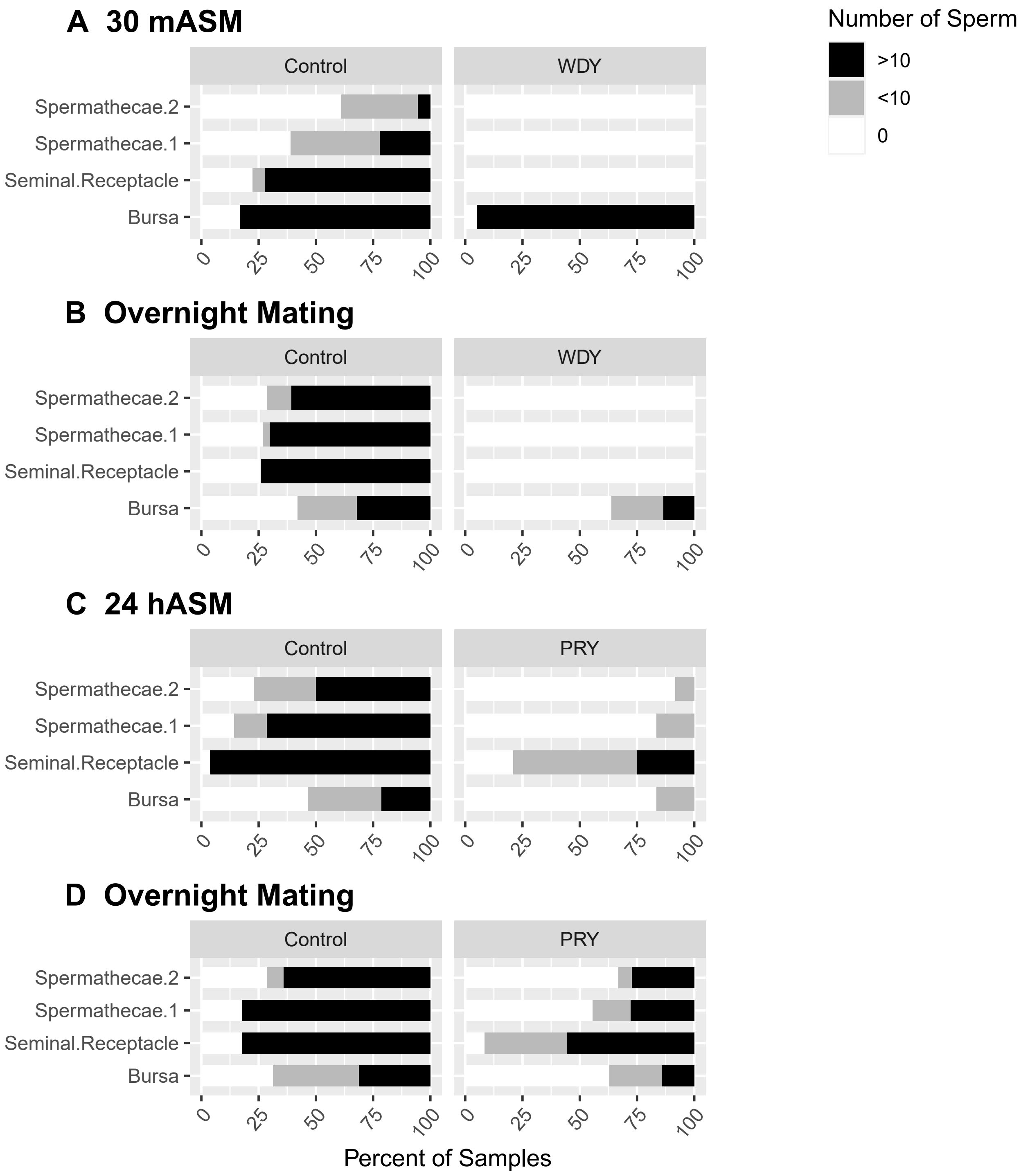

Supplement: Supplementary file 1 [file media-1.pdf]
